# An Approach to Combining Results From Multiple Methods Motivated by the ISO GUM

**DOI:** 10.6028/jres.105.047

**Published:** 2000-08-01

**Authors:** M. S. Levenson, D. L. Banks, K. R. Eberhardt, L. M. Gill, W. F. Guthrie, H. K. Liu, M. G. Vangel, J. H. Yen, N. F. Zhang

**Affiliations:** National Institute of Standards and Technology, Gaithersburg, MD 20899-8120

**Keywords:** Bayes, reference materials, uncertainty

## Abstract

The problem of determining a consensus value and its uncertainty from the results of multiple methods or laboratories is discussed. Desirable criteria of a solution are presented. A solution motivated by the ISO Guide to the Expression of Uncertainty in Measurement (ISO GUM) is introduced and applied in a detailed worked example. A Bayesian hierarchical model motivated by the proposed solution is presented and compared to the solution.

## 1. Introduction

Often a reference material is certified based on data from more than one measurement method (or from more than one laboratory). This situation occurs when no single method can provide the necessary level of accuracy and/or when there is no single method whose sources of uncertainty are well understood and quantified. The intent of using multiple methods is to realize systematic effects (biases) of individual methods as variation across the multiple methods results. The multiple methods should be chosen to avoid common sources of biases, which would invalidate the use of the variation in estimation of the uncertainty of the systematic effects.

If the biases are statistically independent and are centered around zero, then the certified value and the expanded uncertainty can be based on a *t*-interval [1]. Suppose $\bar{X}$ and *s* are the sample mean and sample standard deviation of the results of *n* methods. The interval $\bar{X}\pm t_{n-1,95}s/\sqrt{n}$ is a 95 % confidence interval on the population mean of the methods. Here *t*_*n*−1,95_ is the two-sided 95 percentile point of a *t*-distribution with *n* − 1 degrees of freedom.

There are two problems with the use of the *t*-interval. First, it rests on the assumptions that there is a population of methods whose biases are centered around zero and that the chosen methods are a random sample from the population. Second, when the number of methods is small, the factor *t_n_*_−1,95_ can be very large. For example, if *n* = 2, then *t_n_*_−1,95_ = 12.7 and if *n* = 3, then *t_n_*_−1,95_ = 4.3. For comparison, if *n* is large, the value is close to 2.

**Fig. 1:**
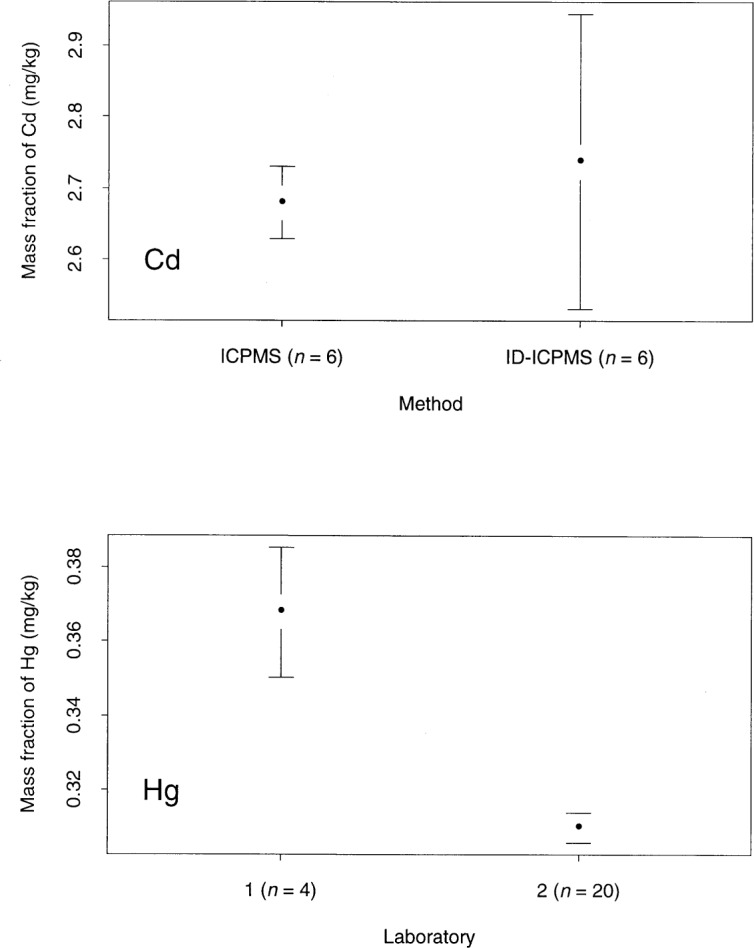
Examples of measurement results. ICPMS means inductively coupled plasma mass spectrometer and ID-ICPMS means isotope dilution inductively coupled plasma mass spectrometry. The numbers in parenthesis are the number of measurements on which the results are based. The uncertainty intervals indicate expanded uncertainties with coverage factors *k* = 2.

To further explore the issues related to the certification from multiple methods, we present an example. Figure 1 summarizes the measurement results of two analytes for a reference material. The analyte Cd was analyzed by two methods. The mean and expanded uncertainty interval (coverage factor *k* = 2) [2,3] of each method are displayed on the top plot. Similarly, the analyte Hg was analyzed by two laboratories and the results are displayed in the bottom plot. In the Cd case, there appears to be agreement between the two methods. It may be reasonable to assume that there are no biases between the two methods.

However, in the Hg case, there appears to be disagreement between the two laboratories. In the certification of this analyte, an uncertainty component for the systematic effects of the laboratories must be considered. The two problems in using a *t*-interval for this uncertainty component, discussed above, are present in the Hg data.

It is the purpose of this paper to propose and justify a solution to the problem of certifying reference materials based on a small number of methods in which the systematic effects are not completely understood. We call this problem the *two-method problem*, although the number of methods may be three or four and laboratories may play the role of methods. Section 2 motivates a set of desirable criteria for a solution and reviews some of the existing solutions to the problem. Section 3 presents a solution, called BOB, based on a Type B model [2,3] of the bias and discusses some implementation issues and related concerns. Section 4 gives a detailed worked example of BOB. Finally, Sec. 5 provides some concluding remarks. Appendix A covers some degrees of freedom issues. Appendix B presents a Bayesian justification of BOB based on a hierarchical model. For a review of the context of the problem in chemical reference materials, see Ref. [4].

## 2. Criteria for a Solution

An important practical property for a solution to the two-method problem is that it is flexible enough to handle a wide variety of settings in a straightforward way. The variety of settings includes the following: (1) the existence and nonexistence of systematic effects in the methods; (2) the availability of two to four methods or laboratories and (3) the existence and nonexistence of a valid uncertainty evaluation for each method (i.e., within-method uncertainty). The alternatives in setting (1) are exemplified by the Cd and Hg results shown in Fig. 1. The Hg results are also relevant to setting (3). In this study, based on knowledge of the laboratories, there is reason to believe that the expanded uncertainty for Laboratory 2 is not valid.

A property often considered desirable for a solution is that it should produce an expanded uncertainty interval that contains the measurement result of each of the methods. The justification for this property is that any of the methods may be the “correct” one since the biases are unknown. From a statistical point of view, this property is not necessary. Statistically, one requires that the expanded uncertainty interval is believed to include the unknown value of the quantity being measured (i.e., measurand [5]) with a stated level of confidence. Under the assumptions described in Sec. 1, the *t*-interval has the correct level of confidence. However, as stated above, if the number of methods is small, the interval may be impractically large.

The solution should possess certain continuity and scaling properties. For example, if the solution has been applied in the two-method case and a third method becomes available, then the result should not change by a large amount. Related to the setting (1) described above, the result should not change abruptly as the systematic effect goes to zero.

In the interest of consistency with current international practice, the solution should not be at odds with the ISO uncertainty guidelines (ISO GUM) [2,3]. Briefly, the ISO guidelines involve expressing the measurement result as a function of quantities whose uncertainties can be evaluated. The uncertainties of these quantities are expressed as standard uncertainties, which are propagated to derive the standard uncertainty of the measurement result. The notation *u*(*X*) is used for the standard uncertainty of the quantity *X*. Along with the standard uncertainties are associated degrees of freedom, which are propagated by the Welch-Satterthwaite formula [2,3]. From the degrees of freedom, a coverage factor *k* is determined based on the *t*-distribution. The expanded uncertainty is equal to the product of the standard uncertainty and the coverage factor, resulting in an interval with a given level of confidence. Often the degrees of freedom are large enough simply to use a coverage factor of *k* = 2.

Finally, the solution should be based on a rigorous statistical model. A statistical model grounds the solution on a strong base. The formulation of such a model clarifies the assumptions of the solution. It also makes available a large literature of properties and results. Appendix B addresses this issue.

Before moving on to the proposed solution, we review currently available procedures. The *t*-interval approach has already been discussed. It has most of the above properties. However, as mentioned above, it depends on assumptions that may not be valid and may produce impractically large intervals when there are a small number of methods. Any similar procedure that estimates the uncertainties associated with the systematic effects of the methods based solely on the observed data will suffer from the same problems. This constraint was one of the guiding principles in the derivation of the proposed solution.

The Schiller-Eberhardt procedure [6] has been used for some time with acceptable results. It is motivated by the desire for the expanded uncertainty interval to contain each of the individual method means. It does not fit into the ISO guidelines and is not based on a rigorous statistical model. It has an undesirable scaling property in that the uncertainty can only increase as the number of methods increases.

Paule-Mandel [7] was developed as an ad hoc procedure to produce a summary value of results from methods with differing biases and precisions. Recently, it has been given a firmer statistical foundation [8]. However, there are unresolved issues related to the uncertainty of the estimate. Additionally, it emphasizes methods with high precision. High precision does not imply low bias.

One final “solution” is to not combine the results if there is an indication of systematic effects that are not understood.

## 3. Type B Model of Bias

In this section, we present a framework for a solution to the two-method problem. The framework is expressed in terms of the language of the ISO guidelines. The model has two components. The first component is the estimate of the population mean of the multiple methods. The second component is the deviation of this population mean from the unknown value of the measurand, i.e., the unknown bias of the population mean. The possible bias is modeled via a Type B distribution [2,3]. (The name BOB comes from Type B On Bias). Type B distributions present a means of incorporating the available information on the problem. Because they are distributions, they can account for uncertainty in the information. Distributional forms should be chosen that capture the information in an effective and straightforward way. These aspects will become more apparent in the specifics that follow.

The measurement model is given by

γ=μ+β, (1)

where *γ* is the unknown value of the measurand, *μ* is the equally weighted mean of the population means of the methods, and *β* is the possible bias of *μ* as an estimate of *γ*. We define *μ* as an equally weighted mean, because in the majority of reference material applications, it is difficult to quantify the relative biases of the the methods. (Greek symbols are used here to emphasize that the quantities are unobserved and unknown.) Both *μ* and *β* require estimates and uncertainties of these estimates. The natural estimate of *μ* is the sample mean of the set of method results. Standard statistical theory gives the uncertainty of this quantity (see example of Sec. 4). For *β* it is most often the case in the present setting to assume that the best estimate is zero. However, it is recognized that there is uncertainty in the estimate. If the best estimate were not zero, then according to the ISO guidelines the measurement result should be adjusted by the nonzero amount.

**Fig. 2:**
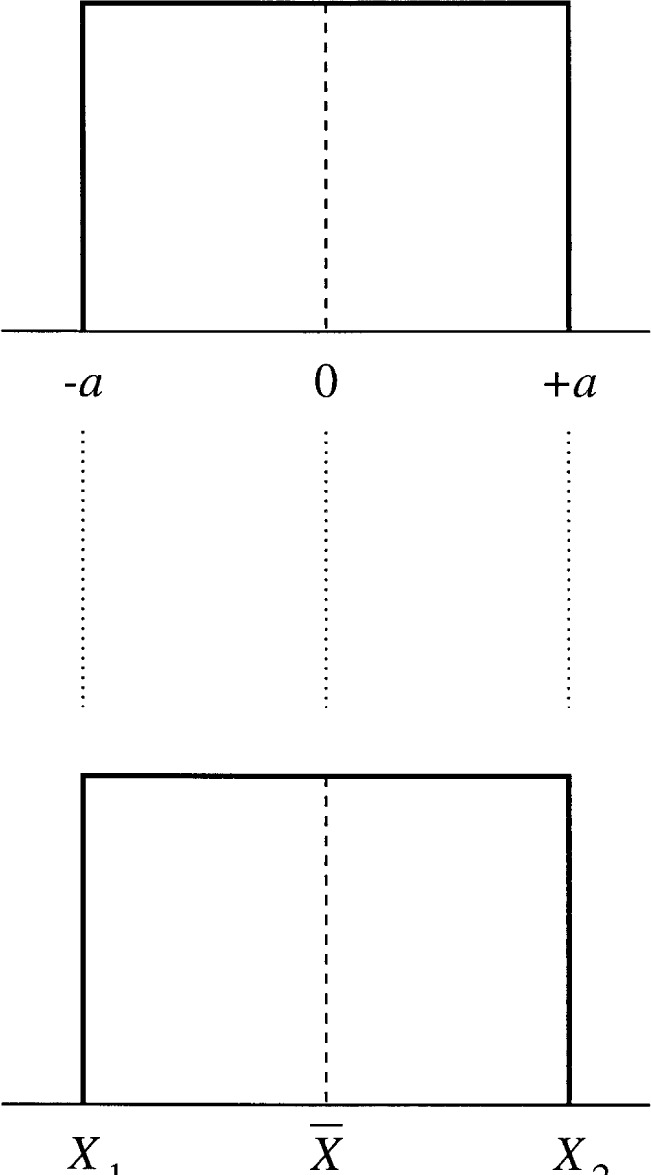
The rectangular (or uniform) distribution.

What is required is a procedure to produce the uncertainty estimate of *β*. To do this, the analyst places a probability distribution on the value *β* that best summarizes the available information. The top plot in Fig. 2 displays a simple and useful distribution for this purpose, called the rectangular (also called uniform) distribution. The distribution models the bias as (1) centered at zero; (2) bounded between ±*a*; and (3) equally likely to be anywhere between ±*a*. Under this assumption, the standard uncertainty of the bias estimate is equal to $a/\sqrt{3}$.

The bottom plot in Fig. 2 in conjunction with the top plot justifies a reasonable choice of *a*. Here the *X*_1_, *X*_2_, and $\bar{X}$ represent, respectively, the results of the two methods and the mean of the two results. Thus, *a* is equal to (*X*_2_ − *X*_1_)/2. Under the measurement model of Eq. (1), this choice of *a* is equivalent to saying that the unknown value of the measurand is believed to be (1) centered at the mean of the two method results; (2) bounded between the two method results; and (3) equally likely to be anywhere between the two method results.

**Fig. 3:**
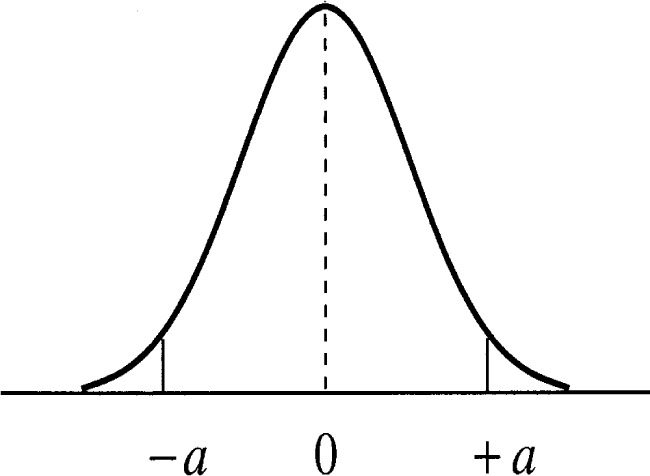
The normal distribution.

There are other useful Type B distributions that can be placed on the bias. Another simple distribution is the normal distribution (see Fig. 3). The normal distribution places higher probability on values near the center of the distribution than values far from the center. It is also unbounded meaning that unlike the rectangular distribution any value is possible. These qualities are represented by the shape of the distribution. There are several ways of employing the normal distribution. If the analyst believes that there is a 95 % chance that the bias is bounded between ±*a*, then the standard uncertainty of the bias is ±*a*/2. As described above, a reasonable value for *a* is equal to (*X*_2_ − *X*_1_)/2. Note that although the normal distribution is unbounded, the use of it described above results in a smaller uncertainty for the bias than the rectangular assumption described above. It is important to note that in the ISO uncertainty procedure only the standard uncertainty matters and not the actual form of the distribution.

### 3.1 Implementation Issues

The previous section described the general framework of the proposed solution to the two-method problem. This section discusses some specific details and implementation issues that will arise in application. We emphasize that although the use of the rectangular distribution was highlighted in the last section as a model for the possible bias, other distributions may be used in the general framework of BOB. The particular distribution is best determined by the experimenter based on the knowledge of the measurement process, previous examples, or assistance from a statistician experienced in the area.

**Fig. 4:**
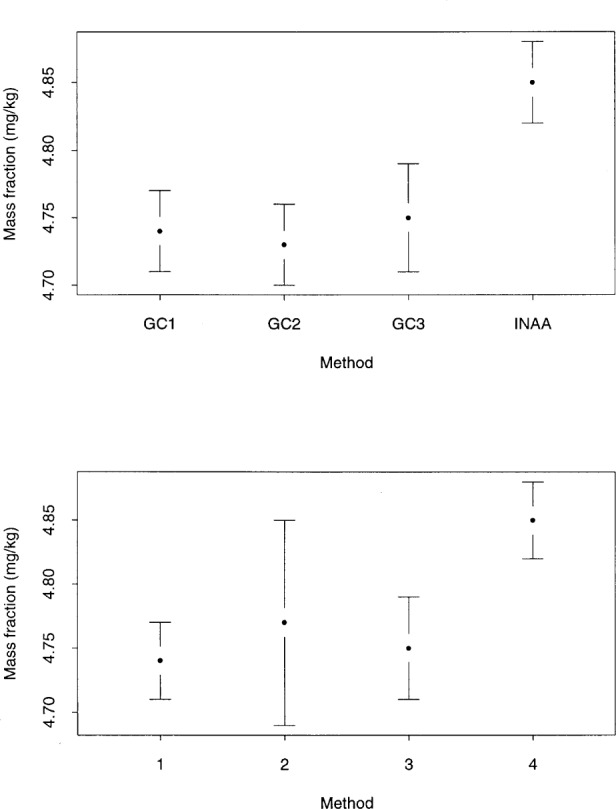
Multimethod examples. GC1, GC2, and GC3 represent gas chromatography using three different columns. INAA means instrumental neutron activation analysis. The uncertainty intervals indicate expanded uncertainties with coverage factors *k* = 2.

Often when there are multiple methods used, the methods are related. The top plot of Fig. 4 illustrates such a situation. There are four methods, but three of the four are related to each other. In this example, three of the methods are gas chromatography (GC) analyses and the forth method is neutron activation (INAA). It is likely that the three GC analyses are more related to each other than to the INAA analysis. The naive use of the *t*-interval approach would be misleading because these are not four independent methods. One procedure for handling this case is to combine the three GC results into a single GC result with an associated uncertainty. Using the combined GC result and the INAA result, the analyst can apply the Type B modeling described in this paper.

The Cd results of Fig. 1 display another important case. In this case, there does not appear to be a between-method effect. The question arises when to apply the procedures described in this paper and when one can assume that there is not a between-method effect. One way of answering this question is to perform a *t*-test (or an *F*-test if the number of methods is greater than 2) on the difference between the two results [1]. The *t*-test, as typically employed with an *α*-level of 0.05, may favor the conclusion that there does not exist a between-method effect. This conclusion may result in underestimating the uncertainty. We recommend that if the *t*-test is used, that the analyst use an *α*-level of 0.5. Alternatively, the use of BOB with the rectangular distribution, as described above, may be effective. If there is not a between-method effect, then the results of the multiple methods should tend to be close to each other. In such a case the width of the distribution on the bias (and its uncertainty) will be small. Thus, there will be little penalty for including the effect when it is small.

The last case we consider is displayed in the bottom plot of Fig. 4. Here the result of Method 1 (represented by the dot) has the lowest value among the four methods. However, the expanded uncertainty interval of Method 2 extends below the intervals of the other three methods. In this case it may make more sense to define the Type B distribution of the bias based on the limits of the expanded uncertainties. In Appendix A, the presence of large within-method uncertainties is addressed with degrees of freedom considerations.

## 4. Example

This section presents a worked example that displays the details of the BOB procedure using the rectangular distribution. The example is based on the Hg data discussed in the body of the paper.

Before starting the example, we review some necessary statistical results. Suppose *W*_1_, *W*_2_,⋯, *W_n_* are *n* independent measurements. Let $\bar{W}$ and s(*W*) denote the sample mean and sample standard deviation, respectively. The standard uncertainty of a sample mean, from the random variation in the measurements, is equal to

$$s(W)/\sqrt{n}.$$ (2)

The associated degrees of freedom for this uncertainty is *n* − 1. In addition to the uncertainty from the random variation, there may exist uncertainty from systematic effects.

We will make multiple uses of the linear measurement equation given by

Y=aW+bZ, (3)

where *a* and *b* are fixed constants with no uncertainty and *W* and *Z* are quantities with uncertainty. Let the standard uncertainties of *W* and *Z* be *u*(*W*) and *u*(*Z*) and the associated degrees of freedom *v_W_* and *v_Z_*. In all that follows, assume that *W* and *Z* are independent. From propagation of uncertainties [2,3], the standard uncertainty of *Y* is equal to

$$u(Y)=\sqrt{a^{2}u^{2}(W)+b^{2}u^{2}(Z)}.$$ (4)

The associated degrees of freedom derived from the Welch-Satterthwaite formula [2,3], is

$$v_{Y}=\frac{u^{4}(Y)}{a^{4}u^{4}(W)/v_{W}+b^{4}u^{4}(Z)/v_{Z}}.$$ (5)

Returning to the example, Table 1 gives the relevant summary statistics for the results from the two laboratories. For notation, let ${\bar{X}}_{1}$, *s*_1_(*X*), and *n*_1_ be the summary statistics for Laboratory 1 and likewise, ${\bar{X}}_{2}$, *s*_2_(*X*), and *n*_2_ be the summary statistics for Laboratory 2. In order to make certain relationships explicit, we use the notation *X*_1_ and *X*_2_ to refer to the two laboratory results including all corrections.

Laboratory 1, in addition to the measurement variation, has a possible systematic effect. The uncertainty of the effect is quantified as a Type B source of uncertainty, referred to as *u*(*S*_1_). We assume that this uncertainty has infinite degrees of freedom. If it were possible to identify all the systematic effects in each laboratory’s measurement process and quantify the respective uncertainties then there would be no need to use the BOB procedure.

Note in the following calculations, many more digits are maintained in the intermediate steps than are shown. This will lead to apparent discrepancies in the equations that follow, in which only a small number of digits are displayed.

### Step 0: The Measurement Equation

The measurement equation model is given by Eq. (1), repeated below:

γ=μ+β, (6)

where *γ* is the unknown value of the concentration, *μ* is the equally weighted mean of the population means of the methods, and *β* is the bias of *μ* as an estimate of *γ*. Each quantity in the model must be estimated. (We use Latin letters to distinguish the estimates, which are observable, from the unobservable unknown values. Uncertainties will be associated with the estimates, as opposed to the unknown values.) The measurement equation relating the estimates is

Y=X+B, (7)

where *Y* is the final measurement result, *X* is the sample mean of *X*_1_ and *X*_2_, and *B* is equal to zero. The final measurement result is

$$\begin{array}{c} Y=X+B=\frac{1}{2}(X_{1}+X_{2})\\ +0=\frac{1}{2}(0.368+0.310)\;\text{mg}/\text{kg}=0.339\;\text{mg}/\text{kg}. \end{array}$$ (8)

We point out here that although the number of measurements for the two methods are not the same, we weight the results equally because there is no reason to believe one result is more accurate than the other. The next steps are the calculation of the uncertainties of *X* and *B* and their combination to obtain the uncertainty of *Y*.

### Step 1: Within-Method Uncertainty

For each laboratory result, calculate the standard uncertainty. For Laboratory 2, the laboratory result is $X_{2}={\bar{X}}_{2}$. The standard uncertainty *u*(*X*_2_) is given by the result for the sample mean [see Eq. (2)]. It is equal to

$$\begin{array}{c} u(X_{2})=u({\bar{X}}_{2})=s_{2}(X)/\sqrt{n_{2}}=\frac{0.0086}{\sqrt{20}}\text{mg}/\text{kg}\\ =0.0019\;\text{mg}/\text{kg}. \end{array}$$ (9)

and the degrees of freedom is equal to $v_{X_{2}}=20-1=19$.

For Laboratory 1, the Type B uncertainty associated with the systematic effect must be included in the uncertainty. The systematic effect is assumed to be an additive effect. The resulting measurement equation is

$$X_{1}={\bar{X}}_{1}+S_{1},$$ (10)

where *S*_1_ is a correction that accounts for the possible systematic effect. The uncertainty of ${\bar{X}}_{1}$ is equal to $u({\bar{X}}_{1})=s_{1}(X)/\sqrt{n_{1}}=0.011\;\text{mg}/\text{kg}/\sqrt{4}=0.0055\;\text{mg}/\text{kg}$ and has $v_{{\bar{X}}_{1}}=4-1=3$ degrees of freedom. Although *u*(*S*_1_) is non-zero, the best estimate of *S*_1_ is zero. Using the results of Eqs. (3)–(5), with *a* = *b* = 1 and $W={\bar{X}}_{1}$ and *Z* = *S*_1_, the standard uncertainty of the Laboratory 1 result is

$$\begin{array}{c} u(X_{1})=\sqrt{u^{2}({\bar{X}}_{1})+u^{2}(S_{1})}=\sqrt{0.0055^{2}+0.006^{2}}\;\text{mg}/\text{kg}\\ =0.0081\;\text{mg}/\text{kg}, \end{array}$$ (11)

with associated degrees of freedom

$$\begin{array}{c} v_{X_{1}}=\frac{u^{4}(X_{1})}{u^{4}({\bar{X}}_{1})/v_{{\bar{X}}_{1}}+u^{4}(S_{1})/v_{S_{1}}}=\frac{0.0081^{4}}{0.0055^{4}/3+0.006^{4}/\infty }\\ =14.4. \end{array}$$ (12)

Note that the term 0.006^4^/∞ is equal to zero. Table 2 summarizes the within-laboratory uncertainties and degrees of freedom.

### Step 2: Between-Method Uncertainty

In the BOB procedure, a Type B distribution is used to account for the possible bias *B* in the average of the results of the methods. In this example, we use the rectangular distribution bounded by the two laboratory results for *B*, as described in Sec. 3, for this purpose. The standard uncertainty based on this distribution is equal to

$$\begin{array}{c} u(B)=\frac{\left|X_{1}-X_{2}\right|}{2\sqrt{3}}=\frac{\left|0.368-0.310\right|}{3\sqrt{3}}\;\text{mg}/\text{kg}\\ =0.0167\;\text{mg}/\text{kg}. \end{array}$$ (13)

Using Eq. 20 of Appendix A, the degrees of freedom for this quantity is

$$\begin{array}{c} v_{B}=\left(\frac{1}{2}\right)\frac{{\left(X_{2}-X_{1}\right)}^{2}}{u^{2}(X_{1})+u^{2}(X_{2})}=\left(\frac{1}{2}\right)\frac{{\left(0.368-0.310\right)}^{2}}{0.0081^{2}+0.0019^{2}}\\ =24.0. \end{array}$$ (14)

### Step 3: Combining Uncertainties

First, we calculate *u*(*X*). Recall $X=\frac{1}{2}(X_{1}+X_{2})=\frac{1}{2}X_{1}+\frac{1}{2}X_{2}$.

Using Eqs. (3)–(5), with *a* = *b* = 1/2,

$$\begin{array}{c} u(X)=\sqrt{{\left(\frac{1}{2}\right)}^{2}u^{2}(X_{1})+{\left(\frac{1}{2}\right)}^{2}u^{2}(X^{2})}\\ =\sqrt{\frac{1}{4}0.0081^{2}+\frac{1}{4}0.0019^{2}}\;\text{mg}/\text{kg}=0.0042\;\text{mg}/\text{kg} \end{array}$$ (15)

and the degrees of freedom of *u*(*X*) is equal to

$$\begin{array}{c} v_{X}=\frac{u^{4}(X)}{{\left(\frac{1}{2}\right)}^{4}u^{4}(X_{1})/v_{X_{1}}+{\left(\frac{1}{2}\right)}^{4}u^{4}(X_{2})/v_{X_{2}}}\\ =\frac{0.0042^{4}}{{\left(\frac{1}{2}\right)}^{4}0.0081^{4}/14.4+{\left(\frac{1}{2}\right)}^{4}0.0019^{4}/19}=16.0. \end{array}$$ (16)

Finally, from the measurement equation, Eq. (7),

$$\begin{array}{c} u(Y)=\sqrt{u^{2}(X)+u^{2}(B)}=\sqrt{0.0042^{2}+0.0167^{2}}\;\text{mg}/\text{kg}\\ =0.017\;\text{mg}/\text{kg} \end{array}$$ (17)

and the corresponding degrees of freedom is equal to

$$\begin{array}{c} v_{Y}=\frac{u^{4}(Y)}{u^{4}(X)/v_{X}+u^{4}(B)/v_{B}}\\ =\frac{0.017^{4}}{0.0042^{4}/16.0+0.0167^{4}/24.0}=27.0. \end{array}$$ (18)

The final summary value and its standard uncertainty for the results of the two-laboratory study are 0.339 mg/kg and 0.017 mg/kg. The degrees of freedom is 27. The multiplier for a 95 % level of confidence interval is 2.1, which is based on a *t*-multiplier with 27 degrees of freedom (see Table B.1 of Ref. [3]). The expanded uncertainty is equal to (2.1)(0.017) mg/kg = 0.036 mg/kg.

## 5. Conclusion

It was stated in Sec. 2 that a guiding principle in the derivation of BOB was the constraint that solutions that are based solely on the observed results will produce intervals whose widths are comparable to the *t*-interval with one degree of freedom, i.e., very large. In other words, two disparate methods give you effectively only two observations of information. BOB does not pull any more information out of the data. BOB overcomes the limitation by bringing in outside information about the measurement processes and quantifying this information in terms of a Type B distribution. The particular distribution is best determined by the experimenter based on the knowledge of the measurement process, previous examples, or assistance from a statistician experienced in the area. In any given application, a reviewer of the uncertainty may disagree with the result. However, in BOB, the outside information appears explicitly and concretely and is open to evaluation. We believe this explicitness, which Bayesian approaches share, is a major strength of BOB.

BOB also possesses many of the desirable criteria discussed in Sec. 2. In particular, it fits in the ISO framework, it is simple to implement, and it is related to a rigorous statistical model (see Appendix B).

## Figures and Tables

**Fig. 5:**
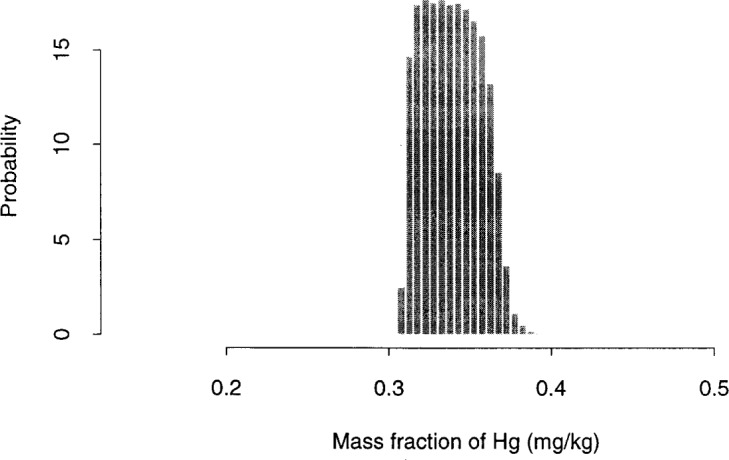
Simulated posterior distribution from Hg data.

**Table 1:** Summary statistics for Hg results

Lab	1	2
${\bar{X}}_{i}$	0.368 mg/kg	0.310 mg/kg
*s_i_*(*X*)	0.011 mg/kg	0.0086 mg/kg
*n*	4	20
*u*(*S_i_*)	0.006 mg/kg	

**Table 2:** Within-method uncertainties

Lab	1	2
*u*(*X_i_*)	0.0081 mg/kg	0.0019 mg/kg
$v_{X_{i}}$	14.4	19

## 6. Appendix A. Degrees of Freedom

The lower plot of Fig. 4 displays an example in which one of the within-method uncertainties is very large. In the basic use of the rectangular distribution presented, the values of the multiple method results are the input into the uncertainty evaluation, that is, $u(B)=|X_{2}-X_{1}|/\sqrt{12}$. If these method results have large uncertainties, the uncertainty evaluation of the possible bias may not be reliable. Degrees of freedom may be used to overcome this problem. Degrees of freedom can be thought of as the uncertainty in the uncertainty. Low degrees of freedom correspond to high uncertainty in the uncertainty. Formula G.3 of Ref. [2] provides an approximation to the degrees of freedom of an estimated standard uncertainty. Using this formula for $u(B)=|X_{2}-X_{1}|/\sqrt{12}$, the degrees of freedom is

$$\left(\frac{1}{2}\right)\frac{{\left(X_{2}-X_{1}\right)}^{2}}{u^{2}(|X_{1}-X_{2}|)}.$$ (19)

We suggest the use of the approximation *u*^2^(|*X*_2_ − *X*_1_|) ≈ *u*^2^(*X*_1_) + *u*^2^ (*X*_2_). Using this approximation, the degrees of freedom is equal to

$$\left(\frac{1}{2}\right)\frac{{\left(X_{2}-X_{1}\right)}^{2}}{u^{2}(X_{1})+u^{2}(X_{2})}.$$ (20)

The approximation is good when |*X*_2_ − *X*_1_| is large relative to *u*(*X*_1_) and *u*(*X*_2_). Under this condition, |*X*_2_ − *X*_1_| is equal to *X*_2_ − *X*_1_ with high probability or equal to *X*_1_ − *X*_2_ with high probability. If the condition is not true the approximation may be poor. Also, when the condition is not met, the use of the approximation will result inappropriately in very small degrees of freedom. We recommend that the degrees of freedom for the bias be at least 3. A value of 3 is equivalent to a 42 % uncertainty in the uncertainty of the bias term. If *X*_1_ and *X*_2_ are normal, an exact formula for *u*^2^(|*X*_2_ − *X*_1_|) is possible based on the folded normal distribution [9].

## 7. Appendix B. Bayesian Model

This appendix presents a Bayesian justification for the BOB procedure. It is more technical than the rest of the paper and uses standard notation for Bayesian statistics. See Ref. [10] for an introduction to Bayesian statistics and the notation used in this section.

Let $\left[{\bar{x}}_{1},s_{1}(x),n_{1}\right]$ and $\left[{\bar{x}}_{2},s_{2}(x),n_{2}\right)$ be the summary statistics for the two methods. Let *μ*_1_ and *μ*_2_ be the population means of the two methods. These latter quantities represent the sample means of a conceptually infinite number of measurements. Let *γ* be the unknown value of ultimate interest.

One natural approach would be to build a hierarchical model around the conditional distribution of *μ*_1_, *μ*_2_|*γ*. We do not follow that path here, because the resulting uncertainty in *γ* would reflect the one degree of freedom problem we are trying to escape. Instead, we reverse the situation and build a model around the distribution *γ*|*μ*_1_, *μ*_2_. What this model will imply is that if one knew *μ*_1_ and *μ*_2_, then there is no more information on *γ* in the observed data. In other words, $\left[{\bar{x}}_{1},s_{1}(x),n_{1}\right]$ and $\left[{\bar{x}}_{2},s_{2}(x),n_{2}\right)$ only provide information on *μ*_1_ and *μ*_2_, which in turn provide information on *γ*.

It is up to the scientists to answer the question: If you knew the results of an infinite number of measurements, i.e., *μ*_1_ and *μ*_2_, what is the distribution that reflects the uncertainty in *γ*, the value of interest? In this appendix, we model *p*(*γ*| *μ*_1_, *μ*_2_) as a uniform distribution centered on (*μ*_1_ + *μ*_2_)/2 and with full width |*μ*_1_ − *μ*_2_|.

We use the conjugate normal model with reference priors for the parameters as the models for the results of the two methods. The basic result of the conjugate normal model is $p[\mu_{1}|{\bar{x}}_{1},s_{1}(x)]$ is the distribution of the quantity ${\bar{x}}_{1}+[s_{1}(x)/\sqrt{n_{1}}]t_{n_{1}-1}$, where $t_{n_{1}-1}$ has a *t*-distribution with *n*_1_ − 1 degrees of freedom. A similar result holds for $p[\mu_{2}|{\bar{x}}_{2},s_{2}(x)]$.

With $p[\mu_{1}|{\bar{x}}_{1},s_{1}(x)]$, $p[\mu_{2}|{\bar{x}}_{2},s_{2}(x)]$, and *p*(*γ*|*μ*_1_, *μ*_2_) given, the posterior distribution $p[\gamma |{\bar{x}}_{1},s_{1}(x),{\bar{x}}_{2},s_{2}(x)]$ is completely specified. Since all the components are basic distributions, standard statistical software can be used to simulate from this posterior distribution. Figure 5 shows the resulting posterior distribution for the Hg data of the paper based on a simulation of 10^5^ values. The sample mean and standard deviation from the simulation are 0.339 mg/kg and 0.018 mg/kg, respectively, compared with 0.339 mg/kg and 0.017 mg/kg from the results for the BOB procedure in Sec. 4.

An exact comparison of the mean and uncertainty for the BOB procedure and the Bayesian model is possible. In the following derivations, we suppress the dependence on the observed quantities.

$$E(\gamma )=E[E(\gamma |\mu_{1},\mu_{2})]=E\left(\frac{\mu_{1}+\mu_{2}}{2}\right)=\frac{{\bar{x}}_{1}+{\bar{x}}_{2}}{2}$$ (21)

$$\text{Var}(\gamma )=E[\text{Var}(\gamma |\mu_{1},\mu_{2})]+\text{Var}[E(\gamma |\mu_{1},\mu_{2})]$$ (22)

$$=E\left(\frac{{\left(\mu_{1}-\mu_{2}\right)}^{2}}{12}\right)+\text{Var}\left(\frac{\mu_{1}+\mu_{2}}{2}\right)$$ (23)

$$=\frac{1}{12}[E^{2}(\mu_{1}-\mu_{2})+\text{Var}(\mu_{1}-\mu_{2})]+\frac{1}{4}\text{Var}(\mu_{1}+\mu_{2})$$ (24)

$$=\frac{1}{12}E^{2}(\mu_{1}-\mu_{2})+\frac{1}{3}\text{Var}(\mu_{1}+\mu_{2})$$ (25)

$$=\frac{{\left({\bar{x}}_{1}-{\bar{x}}_{2}\right)}^{2}}{12}+\frac{1}{3}\left[\frac{n_{1}-1}{n_{1}-3}\frac{s_{1}^{2}(x)}{n_{1}}+\frac{n_{2}-1}{n_{2}-3}\frac{s_{2}^{2}(x)}{n_{2}}\right].$$ (26)

The mean from the BOB procedure is identical to that of Bayes model. The variance from BOB is

$$\frac{{\left({\bar{x}}_{1}-{\bar{x}}_{2}\right)}^{2}}{12}+\frac{1}{4}\left(\frac{s_{1}^{2}(x)}{n_{1}}+\frac{s_{2}^{2}(x)}{n_{2}}\right),$$ (27)

which differs from the Bayes model in the second term. Future work will explore the Bayes model and generalizations of it.

## References

[1] Natrella MG (1963). Experimental Statistics.

[2] International Organization for Standardization (ISO) (1993). Guide to the Expression of Uncertainty in Measurement.

[3] Taylor BN, Kuyatt CE (1994). Guidelines for Evaluating and Expressing Uncertainty in NIST Measurement Results.

[4] Schiller SB (1996). Standard Reference Materials: Statistical Aspects of the Certification of Chemical SRMs.

[5] (1993). International Vocabulary of Basic and General Terms in Metrology (second edition).

[6] Schiller SB, Eberhardt KE (1991). Combining Data from Independent Analysis Methods. Spectrochim Acta.

[7] Paule R, Mandel J (1982). Consensus Values and Weighting Factors. J Res Natl Bur Stand (US).

[b8-j54lev] 8A. L. Rukhin, B. J. Biggerstaff and M. G. Vangel, Restricted Maximum Likelihood Estimation of a Common Mean and the Mandel-Paule Algorithm, to be published.

[9] Patel JK, Read CB (1982). Handbook of the Normal Distribution.

[10] Lee PM (1989). Bayesian Statistics: An Introduction.

